# A Functional Orange Juice Fortified with Beetroot By-Products Attenuates Hyperlipidemia and Obesity Induced by A High-Fat Diet

**DOI:** 10.3390/antiox11030457

**Published:** 2022-02-25

**Authors:** Eman M. Abdo, Omayma El-Sayed Shaltout, Salim Ali, Hanem M. M. Mansour

**Affiliations:** 1 Food Science Department, Faculty of Agriculture (Saba Basha), Alexandria University, P.O. Box 21531, Alexandria 21934, Egypt; prof.dr.omimaelsaidshaltout@alexu.edu.eg (O.E.-S.S.); salim_2007@alexu.edu.eg (S.A.); 2 Food Technology Department, Arid Lands Cultivation Research Institute (ALCRI), City of Scientific Research and Technological Applications (SRTA-City), P.O. Box 21934, New Borg El Arab 21934, Egypt; hmahmoud@srtacity.sci.eg

**Keywords:** obesity, high-fat diet, orange, beetroot stems, beetroot leaves, hyperlipidemia, oxidative stress

## Abstract

Obesity is one of the most prevalent non-communicable diseases and is interlinked with incidences of various diseases. By modulating lifestyle and food quality, obesity can be preventable. The present study investigated the ability of a novel functional beverage based on orange juice and beetroot leaf and stem juice in preventing obesity-associated health issues. To achieve this purpose, we determined the nutritive value of juices and tested their ability to prevent the effect of a high-fat diet on a rat model. Adding leaf and stem juice to orange juice increased the total soluble solids/total titratable acidity ratio, which reflects the high acceptability of the blends, and enhanced their nutritive value. All minerals increased in the blends by increasing the leaf and stem juice percentage. Copper was detected only in the juices containing 10–20% leaf and stem juice (0.01–0.11 mg/100 g). Total flavonoids and betalain increased in the blends, reaching 142.02 µg/mL and 1680 µg/mL, respectively. The mixing process synergized the blends’ radicals scavenging activity. The synergic antioxidant effect of orange enriched with 20% leaf and stem juice attenuated the oxidative stress induced by the high-fat diet by recovering catalase and glutathione peroxidase values. It also enhanced liver enzymes and lipid profile. Consequently, enriching orange with leaf and stem juice results in a functional and nutritious beverage that protects against obesity and its associated health issues.

## 1. Introduction

Obesity is one of the growing non-communicable diseases worldwide—it has tripled since 1975. In 2016, the World Health Organization (WHO) reported that 1.9 billion adults were obese, 340 million adults were overweight, and 340 million children and adolescents were overweight [1]. Obesity is associated with lifestyle and food quality [2]. It is considered the main reason for dyslipidemia, type-2 diabetes, cardiovascular disease, inflammation, and non-alcoholic fatty liver disease (NAFLD) [2]. A high-fat diet is strongly associated with developing obesity, dyslipidemia, and oxidative stress, particularly in the liver [3]. Thus, improving food quality could reduce obesity and its associated health consequences.

Consuming a healthy diet that contains sufficient amounts of vegetables and fruits (about 400 g/day) prevents the incidence of non-communicable diseases (NCDs) [4]. Fruits and vegetables contain considerable amounts of phytochemicals, which are responsible for their antioxidant properties, nutritional value, and functional properties [5]. Phenolics and flavonoids in plants play a role as exogenous antioxidants that aid the endogenous antioxidants in preventing the oxidative stress caused by various diseases [3]. Since few people meet the recommended daily intake of vegetables and fruits, including novel healthy, functional, and ready-to-use beverages based on vegetables and fruits in diets could facilitate meeting the recommended daily intake and reduce the prevalence of various diseases.

Mixing juices promotes the sensory attributes, functional properties, and nutritional value of the beverages; for example, adding beetroot juice to orange juice increases the total phenolics, betalain, ascorbic acid, and the antioxidant activity of the juice [6]. Mixing orange juice with Parkia juice enhances its nutritional value as the amounts of protein, ash, crude fibers, fats, carbohydrate, total phenolics, vitamin A, and vitamin C are increased significantly [5]. Orange (*Citrus sinensis*) is used widely for juice preparation due to its high acceptability and its high nutritive value. It is a rich source of citric acid, potassium, amino acids (serine, arginine, and proline) [7], and ascorbic acid [6]. Besides, it contains a considerable amount of flavanones and flavones, mainly hesperidin, which participates in the juice’s taste and appearance [8].

The aerial part of beetroot (leaves and stalks—the non-consumed plant parts—contain massive amounts of phenolics and flavonoids that exhibit a high antioxidant capacity [9]. They contain a high concentration of the flavonoid vitexin-2-rhamnoside (V2R) [2], which represents about 42% of the total detected flavonoids [3]. Using 0.5 g of the dehydrated beetroot stalk and leaves in rats receiving a high-fat diet decreased the fasting glucose and enhanced insulin resistance [2]. Adding dehydrated beetroot stalk and leaves to rats diminished the deteriorating effect of the high-fat diet by reducing cholesterol and LDL [3]. Similarly, beetroot leaf extract has showed a significant effect on breast and liver cancer which could be attributed to vitexin and the phenolic compounds [10]. Thus, beetroot leaves and stems have revealed promising results in terms of health improvement. 

As we know, studies have focused on using beetroot leaves and stalks in the dried or the hydro-alcoholic extract form. So, our study aimed to investigate the effect of mixing the juice extracted directly from beetroot leaves and stems (LSJ) in fortifying orange juice to present a novel functional and nutraceutical beverage. To achieve this purpose, we examined the LS juice’s ability to improve the product’s nutritive value and investigated the product’s ability to combat the damage resulting from consuming a high-fat diet for three months.

## 2. Materials and Methods

### 2.1. Materials

Balady orange (*Citrus sinensis*) and beetroot (*Beta vulgaris* L.) were collected from the local market in Alexandria, Egypt. Chloroform, methanol, sulfuric acid, boric acid, sodium hydroxide, sodium sulfate, copper sulfate, sodium carbonate, and aluminum chloride were obtained from the Aljomhoria company, Alexandria, Egypt. Folin-Ciocalteu reagent, DPPH, ABTS, and phenolics standards were purchased from Merck, Darmstadt, Germany.

### 2.2. Juice Preparation

Oranges were washed and peeled, and the juice was extracted by a fruit juicer (Tornado TJ-1500S, Turkey). The aerial part of the beetroot (stems and leaves) was cut off from the beetroot pulp and washed carefully to remove any residues before extracting the juice with a fruit juicer (Tornado TJ-1500S, Turkey). The yield of the juices was calculated from the following Equation (1):

Yield (mL/100 g) = [(W1 − W2)/W1] × 100(1)

where (W1) is the weight of the raw plant material before juice extraction, and (W2) is the weight of the pomace.

Both juices were mixed in different proportions as follows: 100% orange juice (OJ), 100% beetroot leaf and stem juice (LSJ), orange juice mixed with 5% of beetroot leaf and stem juice (O-LS 5%), orange juice mixed with 10% of beetroot leaf and stem juice (O-LS 10%), orange juice mixed with 15% of beetroot leaf and stem juice (O-LS 15%), and orange juice mixed with 20% of beetroot leaf and stem juice (O-LS 20%) (Table 1). All juices were pasteurized at 80 °C for 10 min before cooling at 4 °C.

### 2.3. Physicochemical Analysis 

The total soluble solids (TSS) were measured by a digital refractometer NR101, and the results were expressed in °Brix. The pH of the juices was measured by a pH meter (Adwa, AD1030, Szeged, Hungary) at 25 °C. Total titratable acidity (TTA) was determined by titrating the diluted juices (1 juice: 10 dH_2_O) with 0.1N NaOH until the pH reached 8.2; the total acidity was expressed as grams of citric acid per 100 mL according to Equation (2) [11].

(2) $$Acidity\;\%=\frac{V\times N\times 0.067\times 100}{W}\;$$

where (*V*) is mL of NaOH, (*N*) is the normality of NaOH, and (*W*) is the weight of the sample.

The ratio of TSS/TTA was calculated for each sample by dividing the total soluble solids (TSS) by the total titratable acidity (TTA) of the same sample. The viscosity of the juices was determined at 25 °C by a (J.P. Selecta, STS-2011, Barcelona, Spain) viscometer. The color of the prepared juice was determined by HunterLab EasyMatch QC, Reston, Virginia, USA, and the data were expressed in L* (brightness/darkness), R* (redness/greenness), and b* (yellowness/blueness).

### 2.4. Proximate Chemical Composition 

Moisture content was determined by drying the prepared juices in a Wt-binder oven at 105 °C until a constant weight was obtained. Ash content was determined by igniting the sample in a muffle at 500 °C until grey ash was obtained [12]. Total fats were determined according to Folch and Stenly [13]. Total protein was evaluated in the prepared juices by the Kjeldahl method [14]. Total carbohydrates in the juices were calculated by difference according to the following Equation (3):

Total carbohydrates (g/100 g) = 100 − [moisture + lipids + proteins + ash] (3)

### 2.5. Minerals Content

The juice samples were digested by heating 5 mL of the juice with 10 mL of concentrated nitric acid for one hour before cooling, filtering, and diluting them up to 50 mL with dH_2_O [15]. A Jenway, Jenway 6405UV/VIS, Stone, Staffordshire, UK spectrophotometer was used to determine the phosphorous content of the digested samples at 405 nm by the Vanadomolybdophosphoric method; the results were expressed as mg monopotassium phosphate/100 g as described by Jackson [16]. Potassium content was measured by the Backman flame photometer [16]. Inductively Coupled Argon Plasma (ICAP 6500 Duo, Thermo Scientific, Gloucester, UK) was used to determine calcium, magnesium, iron, copper, zinc, and manganese content in the digested samples after standardizing by 1000 mg/L of multi-element certified standard solution, Merck, Darmstadt, Germany.

### 2.6. Vitamin B Content

Vitamins B1, B2, B6, B9, and B12 were determined by HPLC (Agilent, Agilent 1260 series, Stevens Creek BLVD, US). The multi-wavelength detector was adjusted at 280 nm. Five microliters of each sample were separated on ZORBAX SB-C8 column (4.6 mm × 150 mm, 5 μm) using a gradient mobile phase consisting of two solvents. Solvents A and B were water with 0.01% TFA (pH 2.9) and methanol, respectively, at a flow rate of 1.5 mL/min. The ratio of A:B was 90%:10%, 70%:30%, 50%:50%, 90%:10%, and 90%:10% after 0, 1, 4, 8, and 10 min, respectively.

### 2.7. Bioactive Components

To determine the bioactive components, the juice samples were centrifuged at 4000 rpm for 20 min and filtered and stored at −18 ± 2 °C until analysis [17].

#### 2.7.1. Total Phenolic Content (TP) 

Phenolics were measured in the juice samples by the Folin-Ciocalteu method, as described by Vodnar et al. [18]. A mixture of 0.2 N Folin-Ciocalteu reagent (1 mL), 7.5% sodium carbonate (800 µL), and 200 µL of the juice samples was incubated at room temperature in the dark for 2 h. The absorbance of the incubated mixture was measured at 760 nm by using a Jenway, Jenway 6405UV/VIS, Stone, Staffordshire, UK spectrophotometer; the total phenolics were expressed in µg gallic acid/mL of the juice. 

#### 2.7.2. Total Flavonoids (TF) 

Flavonoids were determined by the aluminum chloride method as described by Baba and Malik [19]. After incubating a mixture of 1 mL juice, 4 mL dH_2_O, and 0.3 mL NaNO_2_ (5%) for 5 min, 0.3 mL of AlCl_3_ (10%) was added, and the samples were incubated for 6 min. Afterward, 2 mL of NaOH (1 mol/L) was added, and the volume was completed with dH_2_O up to 10 mL. The mixture was then incubated for 15 min, and the absorbance was measured at 510 nm. The total flavonoid value is expressed as µg/mL of catechin.

#### 2.7.3. Betalain Content

The betalain of the juices was extracted by centrifuging the diluted juice (1:30) at 6000 rpm for 20 min [6]. The betalain was determined spectrophotometrically by measuring the absorbance at 353 and 438 nm to estimate the betacyanin and the betaxanthin, respectively. The betalain content (µg/mL) was calculated according to Equations (4) and (5) [20].

(4) $$\mathrm{Betacyanin}/\mathrm{Betaxanthin}\;(µg/\mathrm{mL})=\frac{A\times \mathrm{DF}\times \mathrm{MW}\times V}{\varepsilon L}$$

where (A) is the recorded absorbance, (DF) is the dilution factor, (MW) is the molecular weight of betacyanin (550 g/mol) and betaxanthin (308 g/mol), (V) is the extract volume in ml, (ε) is the extinction coefficient of betacyanin (60,000 L/mol cm) and betaxanthin (48,000 L/mol cm) in water, and (L) is the length of the cuvette path (1 cm).

Betalain (µg/mL) = Betacyanin (µg/mL) + Betaxanthin(µg/mL)(5)

### 2.8. Antioxidant Activity

#### 2.8.1. DPPH Free Radical Scavenging Assay 

A half millilitre of freshly prepared methanolic DPPH (0.3 mM) was mixed with 0.5 mL of the juice. After incubating at room temperature for 20 min, the absorbance of the samples and the control (DPPH methanolic solution) was measured at 517 nm. The inhibition ratio % was calculated according to Equation (6) as follows:

% Inhibition = (absorbance of control − absorbance of sample/absorbance of control) × 100 (6)

#### 2.8.2. ABTS Radical Scavenging Activity

Two equal portions of previously prepared ABTS solution (7 mmol/L) and potassium persulfate (2.4 mmol/L) were mixed and incubated for 12–16 h in the dark at room temperature until forming a dark greenish-blue solution. One milliliter of the reagent was diluted with 60 mL of dH_2_O to reach 0.701 ± 0.01 at 734 nm. After incubating 10 µL of sample and 4 mL of the reagent for 6 min, the absorbance was measured compared to the control sample [21]. The ABTS radical scavenging activity was calculated from the following Equation (7):

ABTS radical scavenging % = [(absorbance of control − absorbance of sample)/absorbance of control] × 100(7)

### 2.9. Phenolics Profile 

To identify the phenolics in beetroot LS juice, an Agilent, Agilent 1260 series, Stevens Creek BLVD, US HPLC was used. LS juice extract (10 μL) was injected into a Kromasil C18 column (4.6 mm × 250 mm, 5 μm). The mobile phase consisted of two solvents: Solvent (A), water, and solvent (B) acetonitrile/trifluoroacetic acid (99.95:0.05; v:v). The elution was programmed at 35 °C with a gradient flow rate 1 mL/min from 0 min to 16 min as follows: 0 min (82% A); 0–5 min (80% A); 5–8 min (60% A); 8–12 min (60% A); 12–15 min (85% A) and 15–16 min (82% A). The phenolics were detected by a multi-wavelength detector at 280 nm.

### 2.10. Sensory Evaluation

Sensory characteristics of taste, flavor, consistency, and overall acceptance were evaluated by 12 panelists (7 females and 5 males) aged 25–60 years old. In transparent plastic glasses, the control beetroot leaf–stem juice followed by randomized, coded orange juice mixed with different percentages of LS juice (from 0% to 20%) were served to the panelists at room temperature. Then, the panelists were asked to assess the different sensory attributes of the juices by recording a score of nine based on a Hedonic scale from 1 to 9 (1 extremely dislike; 9 extremely like) [22].

### 2.11. Feeding Experiment

#### 2.11.1. Experimental Design

The experimental design was approved by the local committee, and the protocol was agreed with the guidelines of the National Institutes of Health (NIH). The diet offered to the animals was prepared as described in Table 2. Thirty albino rats, at four weeks old (51.9 ± 4 g), were obtained from the housing unit of the Institute of Graduate Studies and Research (IGSR), Alexandria University, Alexandria, Egypt. The rats were housed in plastic cages at 22 ± 2 °C, 60 ± 10% of relative humidity, and 12h light/12h dark. Standard diet and water were provided ad libitum. After one week of acclimatization, the rats were divided randomly into five groups of six rats each. All the served juices were preserved at −20 °C in 15 mL screwcap tubes, which were thawed daily to be served orally to the rats. 

Group (1): negative control (control)—fed with 100% standard diet;

Group (2): positive control (control+)—fed with 100% high-fat diet;

Group (3): (HF-O)—fed with 100% high-fat diet and 9 mL/kg/day of orange juice;

Group (4): (HF-LS)—fed with 100% high-fat diet and 9 mL/kg/day of beets leaf–stem juice;

Group (5): (HF-OLS 20%)—fed with 100% high-fat diet and 9 mL/kg/day of orange juice mixed with 20% of beetroot leaf–stem juice.

The weight of rats was recorded weekly to estimate the weight gain percentage. After 12 weeks, the rats were fasted overnight before being sacrificed under ethyl ether anesthesia. The blood was collected in EDTA tubes for the determination of hemoglobin, hematocrit, and red blood cells, and in non-EDTA tubes for lipid profile, liver enzymes, malondialdehyde, and antioxidant enzymes analysis. 

#### 2.11.2. Weight Gaining 

The gained weight was calculated in each group according to the following Equation (8):

Weight gaining % = [((final weight − initial weight)/final weight) × 100](8)

#### 2.11.3. Blood Parameters Analysis

Blood in EDTA tubes was used to determine hemoglobin (HG), hematocrit, and red blood cells (RBCs) by using an automated hematology analyzer. 

To separate the plasma, the non-EDTA tubes were centrifuged at 3000 rpm for 15 min. The separated plasma was used to determine liver function biomarkers (aspartate transaminase (AST), alanine transaminase (ALT), and alkaline phosphatase (ALP)); lipid profile biomarkers (cholesterol, triglycerides (TG), low-density lipoprotein (LDL), and high-density lipoprotein (HDL)); and kidney function parameters (creatinine and urea). All blood biomarkers were determined spectrophotometrically according to the instructions accompanying the biodiagnostic kits from the Biodiagnostic “Diagnostic and Research Reagents” company, Dokki-Giza, Egypt. 

Malondialdehyde (MDA) was determined in the serum as a result of lipid oxidation as described by Ohkawa et al. [23]. 

Antioxidant enzymes were determined in the plasma as follows: superoxide dismutase (SOD) according to Paglia and Valentine [24], Glutathione peroxidase (GPx) as described by Nishikimi et al. [25], and catalase according to Aebi [26].

### 2.12. Statistical Analysis

IBM SPSS program 25 was used to analyze the data with the one-way ANOVA test. The significant differences of the means were compared by using Duncan’s test at a confident level of 95% (*p* < 0.05). The obtained data were expressed as mean ± standard deviation (SD).

## 3. Results and Discussion

### 3.1. Physicochemical Analysis

**Figure 1:**
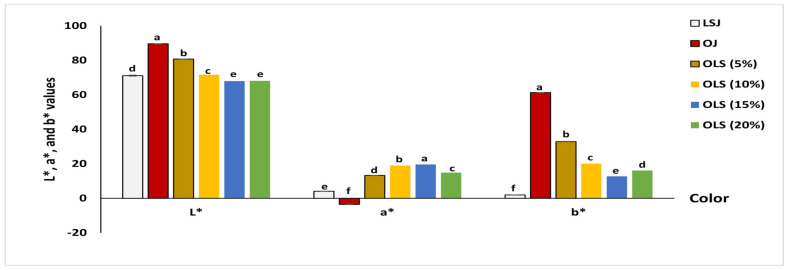
Color of orange juice mixed with beetroot leaf–stem juice in different proportions. Columns in each group labeled with different letters are significantly different at *p* < 0.05.

The physicochemical parameters, including pH, total titratable acidity (TAA), total soluble solids (TSS), viscosity, and the TSS:TAA ratio, are presented in Table 3. In. addition, the color of the blends is illustrated in Figure 1.

The extraction of orange and beet stem and leaf juice yields 64.7% and 60.6% of juice, respectively. Color is one of the most important food characteristics, as it affects the products’ appeal. As illustrated in Figure 1, beetroot leaf and stem juice (LSJ) had values of 4.07 for a* (redness) due to the presence of betacyanin, 1.98 for b* (yellowness) resulting from the betaxanthin, and 71.16 for L* (lightness). Orange juice had values of 89.59, −3.58, and 61.33 for L*, a*, and b*, respectively. The obtained result revealed that beetroot LS juice had a dark red color with some lightness compared to the light-yellow color of orange juice. By increasing the beetroot LSJ added to the orange juice from 5% to 20%, lightness (L*) and yellowness (b*) values decreased by 24% and 80%, respectively, while the redness (a*) of the juices started to increase gradually to reach a value of 19.59. The obtained results agreed with those of Porto et al. [6], who observed that mixing beetroot juice with orange juice increased the redness (a*) of the product.

As demonstrated in Table 3, beetroot LSJ revealed a neutral pH value (6.55), while orange juice is acidic with a pH of 3.58. The obtained pH of orange juice was in the same range as that detected by Porto et al. [6] and Niu et al. [7], but it was lower than the value of 4.35 detected by Adeloye and Agboola [5]. The variation in the pH value could be attributed to the differences among orange varieties. Adding beetroot LSJ to orange juice in a range of 5–20% increased the pH value significantly from 3.81 to 3.96. Porto et al. [6] observed the same result, as the pH value increased by increasing the amount of beetroot juice added to the orange juice. As noticed, the blends remained acidic despite the neutral pH value of the beetroot LSJ. The total titratable acidity (TAA) range of the blends helps to extend the shelf life of the product. As orange juice had the lowest pH value, it had the highest acidity (0.64%), at five times higher than the acidity of beetroot LSJ. The high acidity of orange juice is attributed to the presence of ascorbic acid [5,6]. Adding LSJ to orange juice reduced the acidity value by 20% in OLS (5%) and 40% in OLS (20%). 

By adding beetroot LSJ to orange juice, a significant reduction (nearly half) in the viscosity was observed in the blends compared to orange juice’s viscosity (11.2). Total soluble solids (TSS) is expressed as the °Brix of blends (Table 3). The orange juice had the highest TSS value, at 11.2, which is higher than the value of 5.40 determined by Adeloye and Agboola [5] and that of 8.7–10.8 detected by Niu et al. [7]. We noticed that the TSS of LSJ was three times lower than orange juice, which indicates the low soluble sugars content of LSJ compared to orange juice. The obtained result explained the significant reduction in the TSS value of the blends by increasing the LSJ percentage. 

The total soluble solids: total titratable acidity (TSS:TAA) ratio is a good indicator of the juice’s quality and the consumer acceptability [7]. Juices with TSS:TAA values greater than 20 are considered to be sweet [27]. The TSS:TAA value in orange juice was higher than beetroot LSJ by nearly 37%, which reflects the high acceptability of orange juice. LSJ reduced the TSS: TAA ratio of the blends compared to orange juice. However, by increasing the LSJ percentage from 5% to 20% in the blends, the TSS:TAA value increased from 20.25 to 25.11. The obtained result revealed that adding orange juice to beetroot LS juice increased its acceptability. 

### 3.2. Proximate Chemical Analysis

Beetroot LSJ enhanced the chemical composition of the orange juice slightly (Table 4): we noticed a significant increase in the protein and ash content by 44% and 61%, respectively, in orange juice mixed with 20% of LSJ (OLS 20%) compared to orange juice (OJ). Our results agreed with Adeloye and Agboola [5], who observed that mixing orange juice with parika juice (25:75) increased the protein and the ash content by 89% and 56%, respectively. The increase in the protein content was due to the high protein content of the LSJ. Although the obtained result agreed with the protein content of beetroot leaves as reported by Abdo et al. [9], it was nearly three times higher than the value of 1.8 g/100 g of protein in the dried beetroot leaf and stalk samples reported by Lorizola et al. [28].

Due to the low carbohydrates content of LSJ (7.35%) compared to the value of 19.18% of orange juice, the carbohydrates content decreased gradually in the blends. Carbohydrates in OLS (20%) were roughly 20% lower than orange juice. 

### 3.3. Minerals Content

By increasing the beetroot LS juice percentage added to the orange juice, the minerals content increased significantly (Table 4). Interestingly, although copper was missing in orange juice, we observed copper presence in blends containing 10–20% of LSJ in a range of 0.01–0.11 mg/100 g. The absence of copper in the orange juice disagreed with the results of Niu et al. [7], who observed 0.19–0.78 mg/kg of copper in different orange varieties, which could be attributed to the differences in the agricultural methods and environmental conditions. Copper is a crucial mineral that helps in the metabolism of iron [29] and in turn has a role in anemia prevention. Generally, minerals increased in orange juice containing 20% LSJ compared to orange juice: sodium, magnesium, phosphorous, and potassium increased significantly by 76%, 61%, 41%, and 26%, respectively. Besides these, calcium, iron, and zinc contents were doubled. Sodium and potassium are vital for supporting human health, as studies have investigated their role in blood pressure management and cardiovascular disease treatment [30,31,32]. Similarly, iron is a crucial mineral for anemia treatment and prevention [33].

### 3.4. Vitamin B Content

**Figure 2:**
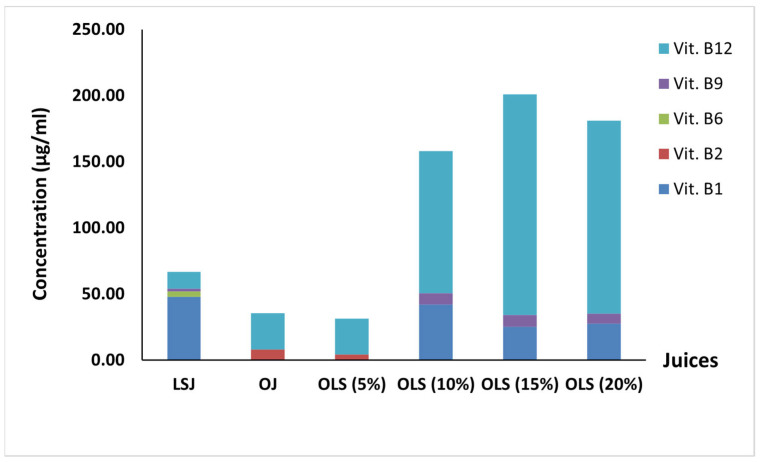
Vitamin B content of orange juice mixed with beetroot leaf–stem juice in different proportions.

B vitamins are a group of eight water-soluble vitamins: thiamine (B1), riboflavin (B2), niacin (B3), pantothenic acid (B5), vitamin B6, folate (B9), and vitamin B12. They play a role as co-enzymes that control the majority of the cellular functions [34]. The B vitamins contents of orange juice, beetroot LS juice, and the blends are shown in Figure 2. We noticed that orange juice is a poor source of B vitamins; it contains only vitamin B12 (27.27 µg/mL) and riboflavin (8.16 µg/mL). On the other hand, riboflavin is missing in beetroot LS juice, and the level of vitamin B12 is about two times lower than in orange juice. However, beetroot LS juice contains 48.08, 3.84, and 2.36 µg/mL of thiamine, vitamin B6, and folate, respectively. By mixing orange with beetroot LS juice, the vitamin content of the resulting blends varied from the original juices. Riboflavin is detected only in OLS (5%) but at nearly half of the concentration in orange juice. Although vitamin B6 was not detected in all the blends, the blends contained a significant amount of vitamin B12, thiamine, and folate. OLS (10%) had the highest thiamin content, at 42.28 µg/mL, and OLS (15%) had the highest vitamin B12 (166.65 µg/mL) and folate (9.07 µg/mL) contents, which were, interestingly, higher than the detected concentrations in both orange and beetroot LS juices. Folate and vitamin B12 intake reduces the risk of neural tube birth defects and hematological disorders, respectively [35]. Therefore, fortifying orange juice with beetroot LSJ increased the B vitamins content, particularly vitamin B12 and folate, which have a crucial role in promoting human health. Besides, these vitamins are vital for the elderly, who suffer from a lack of vitamin B12 absorption in particular.

### 3.5. Bioactive Components

#### 3.5.1. Total Phenolics (TP)

Beetroot LS juice contained the highest phenolic content, at 595.05 µg/mL. However, no significant differences (*p* > 0.05) were noticed in the total phenolic content between orange and beetroot LS juices and the blends. This result disagreed with that of Porto et al. [6], who observed a significant increase in the total phenolics by increasing the beetroot juice added to orange juice, and that of Fernandez et al. [36], who observed a significant increase in the total phenolic content of an orange-based smoothie by adding beetroot leaf ethanolic extract. 

#### 3.5.2. Total Flavonoids (TF)

The total flavonoids content of beetroot LS juice was about 42% higher than orange juice (Table 5). This could be attributed to the high flavonoid content of beetroot leaves and stems [2]. Consequently, adding beetroot LS juice to orange juice in a range of 5–20% increased the total flavonoids by 16–30% in the blends compared to the orange juice.

#### 3.5.3. Betalain

Betalain pigment results from the red-colored betacyanin and the yellow betaxanthin. Betalain is detected only in beetroot LS juice at a concentration of 3860 µg/mL. Hence, adding LS juice increased the betalain in the blends in a range of 1190–1680 µg/mL. Like betalain, betacyanin was increased from 700 µg/mL to 1090 µg/mL by adding the LS juice to the blends. We did not observe an obvious trend regarding the betaxanthin in the blends. However, betaxanthin increased significantly in OLS (20%) compared to OLS (5%). The obtained results agreed with those of Porto et al. [6], who observed an increase in the betalain content by increasing the amount of beetroot juice added to the orange juice. 

### 3.6. Antioxidant Activity

As noticed from Table 5, LS juice had a high antioxidant activity compared to orange juice. A small amount of LS juice (8.21 µg/mL) was able to scavenge 50% of the DPPH^•^ radicals, which is lower than orange juice’s IC_50_ by about 35%. Similarly, the amount of LS juice needed to inhibit the ABTS^•^ activity was lower than orange juice by nearly 20%. The obtained results agreed with those of Porto et al. [6], who observed that beetroot juice has high antioxidant activity compared to orange juice. This result could be due to the high content of betalain and flavonoids in LS juice compared to orange juice. A synergic effect was observed among the blends: by increasing the percentage of LS juice added to orange juice, the antioxidant activity of the blends rose significantly, as 4.05 µg/mL of the OLS (20%) was able to scavenge 50% of the DPPH^•^ radicals. Likewise, adding BLS juice to orange juice increased the antioxidant activity of the blends, as 56.21 µg/mL of OLS (20%) was able to scavenge 50% of ABTS^•^ radicals compared to 59.3 µg/mL of OLS (5%). The obtained results agreed with those of Porto et al. [6] and Adeloye and Agboola [5], who observed a significant increase in the antioxidant activity of orange juice when enriched with beetroot juice and parika juice, respectively. However, our findings disagreed with those of Fernandez, Bengardino et al. [36], who did not detect a significant difference in the DPPH radical scavenging by adding beetroot leaf extract to an orange-based smoothie. 

### 3.7. Phenolics Profile of Beetroot LS Juice

**Figure 3:**
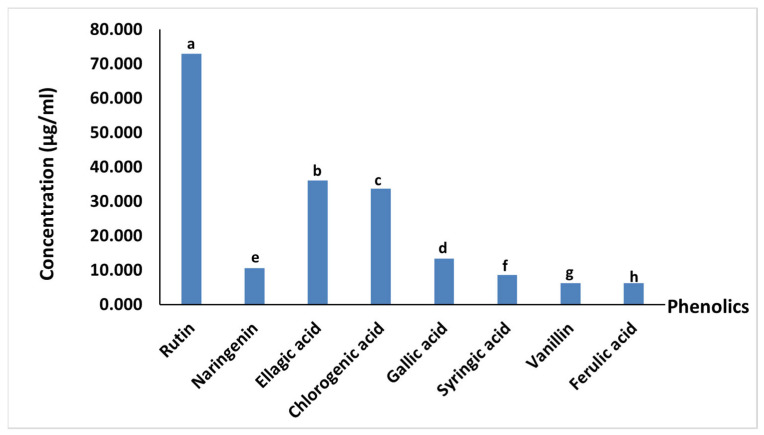
Phenolics profile of beetroot leaf–stem juice. Columns labeled with different letters are significantly different (*p* < 0.05).

In beetroot LS juice, we observed that flavonoids composed nearly half of the phenolics, at about 44.4% of the detected phenolics. Rutin was notably the most common phenolic compound in LS juice. It represented about one-fourth of the detected phenolics. Ellagic and chlorogenic acids ranked second and third after rutin, with percentages of 19.19% and 17.95%, respectively. Besides these, gallic acid, ferulic acid, syringic acid, and vanillin were observed in small amounts ranging from 6.23 µg/mL to 13.46 µg/mL (Figure 3). 

### 3.8. Sensory Evaluation

**Figure 4:**
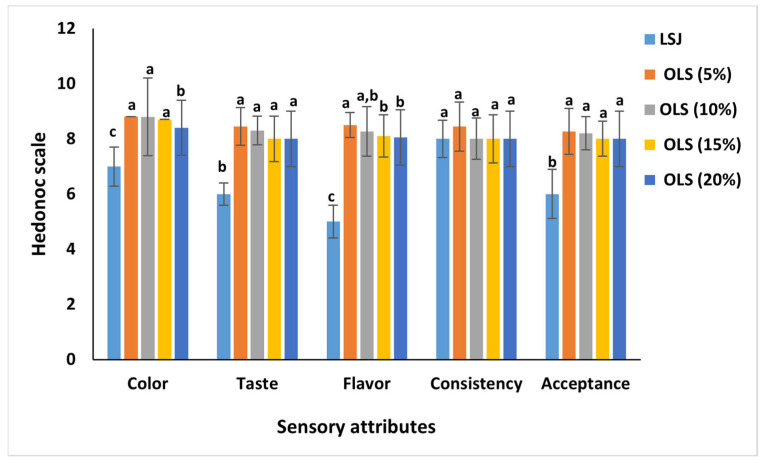
Sensory evaluation of orange juice mixed with beetroot leaf–stem juice in different proportions. Column in each group labeled with different letters are significantly different at *p* < 0.05.

LS juice was found to have the lowest appeal among all panelists in terms of color, taste, flavor, and overall acceptance (Figure 4). However, mixing LS juice with orange enhanced the color, taste, and flavor appeal. The increase in the palatability of the blended juices compared to the LS juice could be attributed to the high organic acids present in orange juice [6], which is the reason that orange juice is used as a base for most juices. Similarly, mixing beetroot juice and parika juice with orange improved the palatability of blends in [6] and [5], respectively. However, Zein, Hashish et al. [10] observed that adding 0.5 g of beetroot powder to the orange juice did not affect the odor but reduced all the other organoleptic characteristics and the product’s acceptability. It should be mentioned that the panelists noted an undesired taste in the blend when increasing the amount of LS juice over 20%. However, no significant differences were observed regarding the sensory attributes of all blends.

### 3.9. Weight Gaining

**Figure 5:**
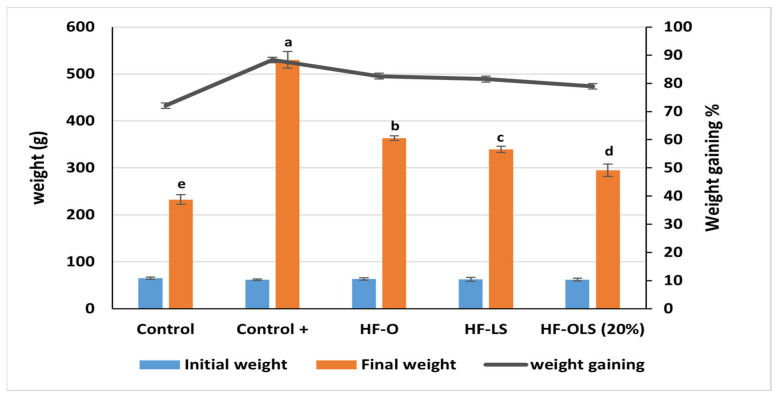
Initial weight, final weight, and weight gain of rats fed with standard diet, high fat diet, and a high fat diet and juice for three months. Control (Rats fed with a standard diet), Control+ (rats fed with a high-fat diet composed by mixing standard diet with 20% of vegetarian ghee), HF-O (rats fed with a high-fat diet and 9 mL/kg/day of orange juice), HF-LS (rats fed with a high-fat diet and 9 mL/kg/day of beetroot leaf–stem juice), HF-OLS (20%) (rats fed with a high-fat diet and 9 mL/kg/day of orange juice mixed with 20% of beetroot leaf–stem juice). Columns of the final weight in feeding each group labeled with different letters are significantly different at *p* < 0.05.

As noticed from Figure 5, the final weight of rats fed with the high-fat diet (control+) was nearly double that of the control group fed with the standard diet. Similarly, Lorizola et al. [3] noticed that feeding rats with a high-fat diet for eight weeks induced obesity in rats, as the rats gained weight by nearly 49% compared to the control [3]. Therefore, the high-fat diet was able to induce obesity in rats. The weight gain of the (control+) group was about 20% higher than the control group. Incorporating orange juice in the HF-O group diet and LS juice in the HF-LS group diet reduced the weight by 36% and 41%, respectively, compared to the (control+) group fed with the high-fat diet. Feeding the rats in the HF-OLS (20%) group with 9 mL/kg/day of orange juice mixed with 20% LS juice showed a synergistic effect in reducing the induction of obesity in the rats by half. The obtained result agreed with that of Lee et al. [37], who observed that involving lyophiliyzed beetroot leaves in rats receiving a high cholesterol diet reduced the gained weight significantly compared to the control. Similarly, Al-Harbi et al. [38] noticed that beetroot stem extract reduced the weight in diabetic rats fed with a high-fat diet.

### 3.10. Hematology Analysis

**Figure 6:**
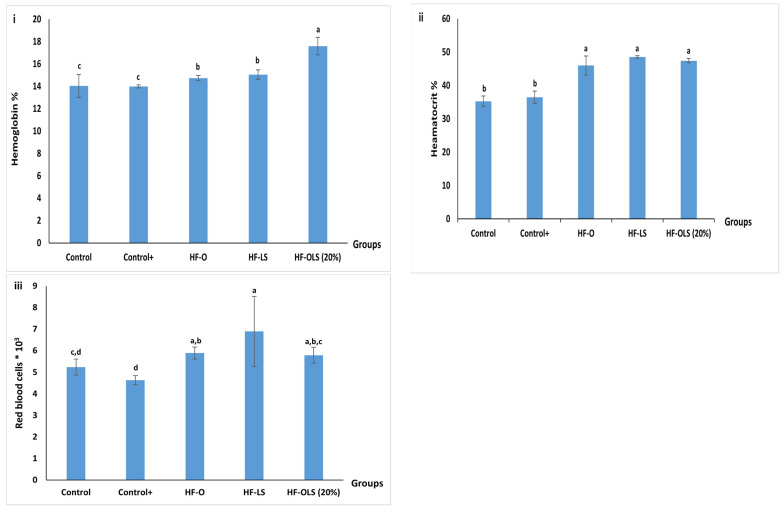
Hematological analysis of rats fed with standard diet, high fat diet, and high fat diet and juice for three months. (**i**): Hemoglobin%, (**ii**): red blood cells (RBCs) *10^3^, and (**iii**): hematocrit%. Control (rats fed with a standard diet), Control+ (rats fed with a high-fat diet composed by mixing standard diet with 20% of vegetarian ghee), HF-O (rats fed with a high-fat diet and 9 mL/kg/day of orange juice), HF-LS (rats fed with a high-fat diet and 9 mL/kg/day of beetroot leaf–stem juice), HF-OLS (20%) (rats fed with a high-fat diet and 9 mL/kg/day of orange juice mixed with 20% of beetroot leaf–stem juice). Columns labeled with different letters are significantly different at *p* < 0.05.

Red blood cells, hemoglobin, and hematocrit content are shown in Figure 6. Incorporating 9 mL/kg/day of beetroot LS juice in the rats’ diet in the HF-LS group peaked the amount of red blood cells (RBCs) compared to the other groups, at 6.89 × 10^3^ (Figure 6ii). On the other hand, feeding rats with 9 mL/kg/day of OLS (20%) juice in the HF-OLS (20%) group elevated the hemoglobin content, causing it to reach a value of 17.6%, which is significantly higher than the other groups (Figure 6i). The obtained result agreed with that of Abdulmaguid [39], who observed that adding beetroot juice to orange–strawberry–mango juice increased the amounts of hemoglobin and the hematocrit significantly compared to consuming orange juice only. Our results agreed with those of Gheith and El-Mahmoudy [40], who reported that administrating beetroot greens (leaf and stalk) extract restored RBCs and hemoglobin content within two weeks. The hemoglobin value detected in the present study was about 12% higher than the hemoglobin content of anemic rats fed with biscuits containing 15% of beetroot pomace, as reported by Abdo et al. [33]. The obtained result could be attributed to the high VB12 of LS juice, which prevents anemia incidence, and the high ascorbic acid content of orange juice, which increases the absorbance of the iron present in the LS juice.

No significant difference (*p* > 0.05) was detected in the hematocrit content among the HF-O, HF-LS, and HF-OLS groups (Figure 6iii). However, the hematocrit concentration among the rats of those groups was significantly higher than the control and the (control+) groups. 

### 3.11. Liver Function Enzymes

**Figure 7:**
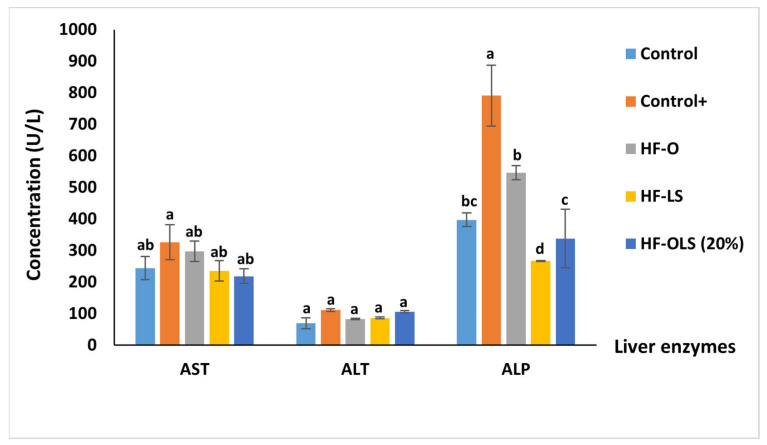
Liver enzymes: AST (aspartate transaminase), ALT (alanine transaminase), and ALP (alkaline phosphatase) of rats fed with standard diet, high fat diet, and high fat diet and juice for three months. Control (rats fed with a standard diet), Control+ (rats fed with a high-fat diet composed by mixing a standard diet with 20% of vegetarian ghee), HF-O (rats fed with a high-fat diet and 9 mL/kg/day of orange juice), HF-LS (rats fed with a high-fat diet and 9 mL/kg/day of beetroot leaf–stem juice), HF-OLS (20%) (rats fed with a high-fat diet and 9 mL/kg/day of orange juice mixed with 20% of beetroot leaf–stem juice). Columns of each enzyme labeled with different letters are significantly different at (*p* < 0.05).

As observed from Figure 7, consuming a high-fat diet for three months affected the liver negatively. The highest values of aspartate transaminase (AST), alkaline phosphatase (ALP), and alanine transaminase (ALT) enzymes were recorded in the control^+^ group compared to the other groups. 

Ingesting 9 mL/kg/day of LS juice in the HF-LS group reduced the ALP significantly by nearly 66% and 51% compared to the control+ and the HF-O groups, respectively. Adding beetroot LS juice to orange juice in the HF-OLS (20%) group nearly halved the ALP value compared to the (control+) group and enhanced the effect of the orange juice on reducing the ALP by 38% compared to the HF-O group. The observed reduction in the ALP could be attributed to the high antioxidant activity of the blends. 

The lowest values of AST and ALT were recorded in the HF-OLS (20%) group. However, apart from the (control+) group, this result had no significant difference from the other groups (*p* > 0.05). The obtained result disagreed with that of Babarykin et al. [41], who reported a significant reduction in the AST and the ALT concentrations after administrating 1 mL of a fractionated beetroot juice to obese rats. 

### 3.12. Kidney Functions

**Figure 8:**
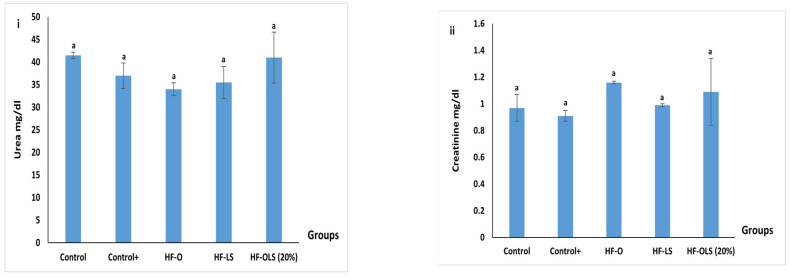
Kidney functions: (**i**) urea (mg/dl) and (**ii**) creatinine (mg/dl) of rats fed with standard diet, high fat diet, and high fat diet and juice for three months. Control (rats fed with a standard diet), Control+ (rats fed with a high-fat diet composed by mixing a standard diet with 20% of vegetarian ghee), HF-O (rats fed with a high-fat diet and 9 mL/kg/day of orange juice), HF-LS (rats fed with a high-fat diet and 9 mL/kg/day of beetroot leaf–stem juice), HF-OLS (20%) (rats fed with a high-fat diet and 9 mL/kg/day of orange juice mixed with 20% of beetroot leaf–stem juice). Columns labeled with different letters are significantly different at *p* < 0.05.

No significant differences (*p* > 0.05) were detected among the groups in terms of urea and creatinine values, as shown in Figure 8. The obtained result agreed with that of Abdo et al. [33], who reported no differences in kidney function parameters after administrating biscuits containing 15% of beetroot pomace to rats for 28 days.

### 3.13. Lipid Profile

**Figure 9:**
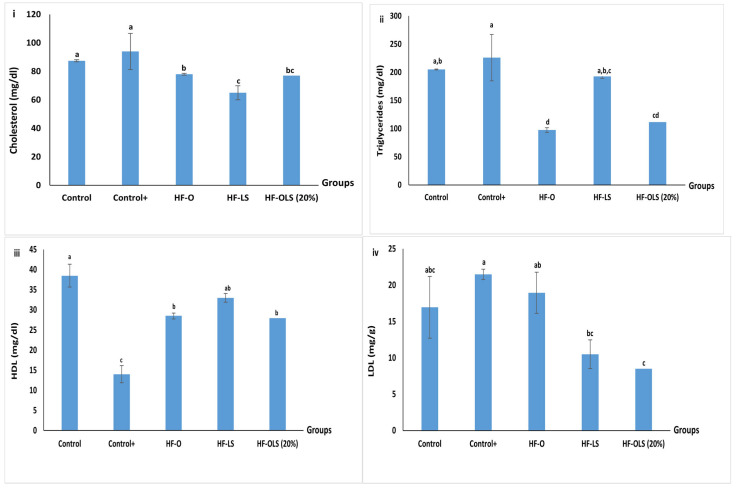
Lipid profile—(**i**) cholesterol (mg/dL), (**ii**) triglycerides (mg/dL), (**iii**) HDL (mg/dL), and (**iv**) LDL (mg/dL)—of rats fed with standard diet, high fat diet, and high fat diet and juice for three months. Control (rats fed with a standard diet), Control+ (rats fed with a high-fat diet composed by mixing a standard diet with 20% of vegetarian ghee), HF-O (rats fed with a high-fat diet and 9 mL/kg/day of orange juice), HF-LS (rats fed with a high-fat diet and 9 mL/kg/day of beetroot leaf–stem juice), HF-OLS (20%) (rats fed with a high-fat diet and 9 mL/kg/day of orange juice mixed with 20% of beetroot leaf–stem juice). Columns labeled with different letters are significantly different at *p* < 0.05.

Enriching orange juice with beetroot LS juice ameliorated the lipid profile of rats fed with a high-fat diet for three months (Figure 9). This could result from the positive effect of beetroot in decreasing the total cholesterol by increasing the HDL [42] and decreasing the LDL [3]. The total cholesterol detected in the control^+^ group was 18% higher than the control group. This result agreed with that of Lorizola et al. [3], who detected a 22% increase in the total cholesterol content of rats fed with a high-fat diet compared to the control. So, the high-fat diet was able to induce metabolic disorders in the rats.

Serving 9 mL/kg of LS juice daily to rats in the HF-LS group decreased the cholesterol significantly by nearly 31% compared to the (control+) group. Similarly, using 1 mL of fractionated beetroot juice reduced the total cholesterol in the obese rats, as reported by Babarykin et al. [41]. Therefore, mixing the LS juice with orange enhanced its ability to decrease the total cholesterol in the HF-OLS (20%) group by 20% compared to the (control+) group. Interestingly, our result was more promising than that of Lorizola et al. [3], who found that using beetroot stalks and leaves in a dried form or as ethanolic extract reduced the total cholesterol in obese rats by 15% and 11%, respectively. In contrast to Lorizola et al. [3], we used the juice extracted from beetroot leaves and stems; the mechanical treatment could release more bioactive components, which have different biological effects.

Similarly, triglycerides and LDL values peaked in the (control+) group fed with a high-fat diet, while the lowest value of HDL content was recorded compared to the other groups. Incorporating 9 mg/kg of beetroot LS juice daily in the diet of rats in the HF-LS group decreased the triglycerides significantly by nearly 57% compared to the (control+) group. Likewise, orange mixed with 20% LS juice reduced the triglycerides in the HF-OLS (20%) group by 50% compared to the (control+) group. The ability of beetroot LS juice to reduce triglyceride was nearly 2 times higher than the reduction observed by Raish et al. [43] when administrating beetroot juice to rats. 

Besides, OLS (20%) recovered the HDL to a value close to the control group (28 mg/dL). Moreover, it showed a potent effect in decreasing LDL by nearly 60% compared to the (control+) group, which agreed with the results reported by Raish et al. [43]. This decrease could be correlated to the synergic effect of the mixture’s antioxidant activity.

### 3.14. Antioxidant Enzymes and Malondialdehyde

**Figure 10:**
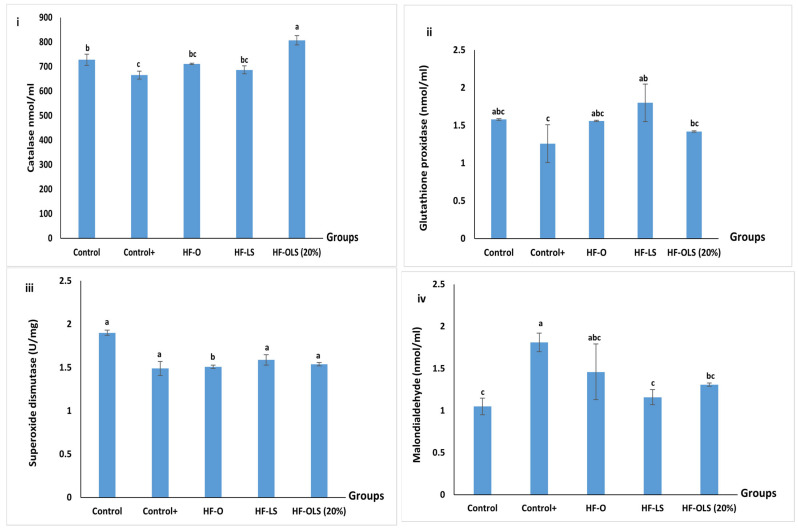
Oxidant and antioxidant enzymes of rats fed with standard diet, high fat diet, and high fat diet and juice for three months. (**i**): catalase (mmol/mL), (**ii**): glutathione peroxidase (mmol/mL), (**iii**): superoxide dismutase (U/mg), and (**iv**): malondialdehyde (mmol/mL). Control (rats fed with a standard diet), Control+ (rats fed with a high-fat diet composed by mixing a standard diet with 20% of vegetarian ghee), HF-O (rats fed with a high-fat diet and 9 mL/kg/day of orange juice), HF-LS (rats fed with a high-fat diet and 9 mL/kg/day of beetroot leaf–stem juice), HF-OLS (20%) (rats fed with a high-fat diet and 9 mL/kg/day of orange juice mixed with 20% of beetroot leaf–stem juice). Columns labeled with different letters are significantly different at *p* < 0.05.

Catalase, superoxide dismutase (SOD), and glutathione peroxidase (GPx) enzymes are naturally created in the body to inhibit oxidant activity. In the present study, those enzymes recorded the lowest concentrations in the control^+^ group compared to the other groups due to the prolonged oxidative stress that occurred in the rats’ bodies (Figure 10). 

Serving 9 mL/kg/day of OLS (20%) juice to the rats ameliorated the oxidative stress induced by the high-fat diet by recovering the catalase and GPx values. 

We noticed a synergic effect of OLS (20%) juice in the HF-OLS (20%) group in recovering the catalase compared to serving orange juice and LS juice to HF-O and HF-LS groups, respectively (Figure 10i). The obtained results agreed with Lorizola et al. [3], who reported that incorporating the dried beetroot leaves and stalk in the rats fed with a high-fat diet enhanced the oxidative stress compared to the rats fed with a high-fat diet only. 

The GPx increased significantly in the HF-OLS (20%) by 11% compared to the (control+) group. Similar to our result, Lee et al. [37] detected an enhancement in the GPx value in rats fed with lyophilized beetroot leaves compared to rats receiving a high cholesterol diet.

On the other hand, the recovery of the SOD in the HF-OLS (20%) group was only by 3% compared to the control^+^ group, which is not a significant amelioration (*p* > 0.05) (Figure 10iii). Our obtained result agreed with those of Lorizola et al. [3], who detected that dehydrated beetroot leaves and stalks did not affect the SOD concentration compared to rats fed with a high-fat diet only, and Lee et al. [37], who observed that beetroot leaves did not enhance SOD activity in rats fed with a high cholesterol diet. However, our findings disagreed with those of Raish, Ahmad et al. [43], who observed a significant amelioration of the isoproterenol-induced oxidative stress by recovering the SOD in rats fed with beetroot juice.

Although the tissues secreted endogenous antioxidant enzymes in the (control+) group, malondialdehyde was 42% higher than in the control group. So, the high-fat diet was able to induce oxidative stress in the rats. Involving LS juice in the rat’s diet reduced the MDA by 36% compared to the control^+^ group, which is in agreement with the findings of Lorizola et al. [3]. As noticed from Figure 10iv, LS juice enhanced the orange juice’s ability to reduce the MDA significantly (*p* < 0.05) by nearly 28% compared to the (control+) group. This reduction is attributed not only to the activity of the antioxidant enzymes but also to the bioactive component present in the new blended juice: flavonoids and betalain. Our product’s ability to prevent lipid peroxidation was lower than the freeze-dried beetroot leaves’ ability detected by Lee et al. [37]. This could be a consequence of the concentration of the bioactive components in the studied plant parts, as lyophilization increased the leaves’ ability to prevent the oxidation process compared to the juice used in the present study.

## 4. Conclusions

Briefly, mixing orange juice with LS juice results in a beneficial effect for both juices. It enhances the orange juice’s nutritive value and improved the palatability of the LS juice. The orange–LS blends share a high protein, minerals, flavonoid, and betalain content. Increasing the amount of LS juice added to the orange juice revealed a synergic effect in scavenging the DPPH and the ABTS radicals that could contribute to enhancing human health. Due to the high-quality nutritive value of the blends, serving 9 mL/kg/day of OLS-20% to rats receiving a high-fat diet for three months boosted the rats’ health significantly. The blend contributed to reducing the gained weight to half, prevented lipid peroxidation and reduced the MDA, recovered the antioxidant enzymes, and ameliorated the lipid profile and the liver functions. Consequently, beetroot leaves and stems should be incorporated in food manufacturing as they lead to promising results for food nutrition enhancement and non-communicable disease prevention.

## Figures and Tables

**Table 1:** The blends of orange juice and beetroot leaf and stem juice.

JUICE	ORANGE JUICE %	BEETROOT LEAF AND STEM JUICE %
**OJ**	100	0
**LSJ**	0	100
**OLS (5%)**	95	5
**OLS (10%)**	90	10
**OLS (15%)**	85	15
**OLS (20%)**	80	20

**Table 2:** Standard and high-fat diet composition (%) “based on the dry weight”.

	Standard Diet	High-Fat Diet
**Dried skim milk**	37.50	37.50
**Corn starch**	33.45	3.45
**Cellulose**	5.00	5.00
**Sucrose**	10.00	15.00
**Corn oil**	9.00	9.00
**Vegetable ghee**	0.00	25.00
**Vitamin mix**	1.00	1.00
**Minerals mix**	4.00	4.00
**Chlorine chloride**	0.02	0.02
**L-cystine**	0.03	0.03
**Total**	100.00	100.00

**Table 3:** Physicochemical properties of orange juice mixed with beetroot stem–leaf juice in different proportions.

	LSJ	OJ	OLS (5%)	OLS (10%)	OLS (15%)	OLS (20%)
**PH**	6.55 ± 0.01 ^a^	3.58 ± 0.42 ^c^	3.81 ± 0.07 ^b^	3.84 ± 0.06 ^b^	3.88 ± 0.03 ^b^	3.96 ± 0.03 ^b^
**TAA %**	0.13 ± 0.02 ^b^	0.67 ± 0.18 ^a^	0.54 ± 0.05 ^a,b^	0.50 ± 0.04 ^a,b^	0.47 ± 0.09 ^a,b^	0.40 ± 0.07 ^b^
**VISCOSITY**	58.35 ± 2.12 ^b^	116.50 ± 0.49 ^a^	57.95 ± 0.08 ^b^	57.00 ± 1.41 ^b^	57.50 ± 0.71 ^b^	56.50 ± 0.71 ^b^
**TSS (°BRIX)**	3.70 ± 0.57 ^d^	11.20 ± 0.14 ^a^	10.80 ± 0.14 ^a^	10.60 ± 0.15 ^a,b^	10.00 ± 0.10 ^b,c^	9.90 ± 0.14 ^c^
**TSS/TAA**	17.38 ± 0.33 ^b^	27.59 ± 4.71 ^a^	20.25 ± 1.88 ^a,b^	21.43 ± 1.36 ^a,b^	21.74 ± 4.09 ^a,b^	25.11 ± 1.08 ^a^

Data are presented as mean ± standard deviation (SD). Mean values in a row with different superscripts are significantly different at *p* < 0.05.

**Table 4:** Proximate chemical composition and minerals content of orange juice mixed with beetroot stem–leaf juice in different proportions.

*Sample*	*LSJ*	*OJ*	*OLS (5%)*	*OLS (10%)*	*OLS (15%)*	*OLS (20%)*
*Proximate chemical composition* (g/100 g)						
*Moisture %*	85.60 ± 0.10 ^a^	79.95 ± 1.02 ^c^	80.21 ± 1.68 ^c^	80.42 ± 0.57 ^c^	83.25 ± 0.35 ^b^	83.09 ± 0.09 ^b^
*Total fats %*	0.52 ± 0.02 ^a^	0.51 ± 0.01 ^a^	0.55 ± 0.06 ^a^	0.53 ± 0.03 ^a^	0.59 ± 0.06 ^a^	0.60 ± 0.10 ^a^
*Total proteins %*	5.20 ± 0.17 ^a^	0.24 ± 0.03 ^c^	0.24 ± 0.00 ^c^	0.26 ± 0.14 ^c^	0.29 ± 0.03 ^c^	0.43 ± 0.04 ^b^
*Ash %*	1.33 ± 0.06 ^a^	0.12 ± 0.03 ^e^	0.20 ± 0.02 ^c,d^	0.15 ± 0.01 ^d,e^	0.25 ± 0.04 ^b,c^	0.31 ± 0.01 ^b^
Carbohydrates % *	7.35 ± 0.26 ^c^	19.18 ± 1.08 ^a^	18.80 ± 1.60 ^a^	18.64 ± 0.59 ^b^	15.62 ± 0.28 ^b^	15.56 ± 0.4 ^b^
*Minerals content* (mg/100 g)						
*Ca*	125.85 ± 3.25 ^a^	14.90 ± 2.95 ^c^	16.90 ± 1.91 ^c^	13.50 ± 1.63 ^c^	27.30 ± 5.90 ^b^	28.60 ± 0.74 ^b^
*Mg*	5.69 ± 0.26 ^c^	7.60 ± 3.29 ^c^	6.60 ± 0.49 ^c^	11.20 ± 1.24 ^b,c^	14.70 ± 1.08 ^b^	19.60 ± 0.99 ^a^
*K*	266.15 ± 4.65 ^a^	53.70 ± 5.10 ^d^	50.10 ± 2.65 ^d^	51.80 ± 0.21 ^d^	64.70 ± 3.06 ^c^	72.70 ± 0.19 ^b^
*Na*	206.70 ± 5.69 ^a^	10.60 ± 2.15 ^c^	14.50 ± 1.2 ^c^	18.10 ± 0.95 ^c^	39.40 ± 8.77 ^b^	44.80 ± 0.62 ^b^
*P*	3.67 ± 0.12 ^c^	19.70 ± 4.62 ^b^	20.90 ± 0.25 ^b^	23.80 ± 4.14 ^a,b^	29.80 ± 7.48 ^a,b^	33.40 ± 0.70 ^a^
*Fe*	1.31 ± 0.04 ^c^	1.60 ± 0.27 ^c^	2.10± 0.01 ^c^	1.98 ± 0.00 ^c^	2.90 ± 0.46 ^b^	3.70 ± 0.08 ^a^
*Mn*	0.53 ± 0.00 ^a^	0.01 ± 0.00 ^b^	0.10 ± 0.12 ^b^	0.03 ± 0.01 ^b^	0.09 ± 0.00 ^b^	0.12 ± 0.01 ^b^
*Cu*	0.62 ± 0.01 ^a^	ND	ND	0.01 ± 0.00 ^d^	0.06 ± 0.02 ^c^	0.11 ± 0.03 ^b^
*Zn*	2.19 ± 0.01 ^a^	0.47 ± 0.04 ^c^	0.51 ± 0.03 ^c^	0.57 ± 0.01 ^c^	0.88 ± 0.16 ^b^	0.94 ± 0.04 ^b^

Data are presented as mean ± standard deviation (SD). Mean values in a row with different superscripts are significantly different at *p* < 0.05. * Carbohydrate content determined by difference, ND (not detected).

**Table 5:** Bioactive components and antioxidant activity of orange juice mixed with beetroot stem–leaf juice in different proportions (µg/mL).

	LSJ	OJ	OLS (5%)	OLS (10%)	OLS (15%)	OLS (20%)
**Total phenolics**	595.05 ± 10.97 ^a^	582.13 ± 44.45 ^a^	540.05 ± 76.76 ^a^	554.21 ± 16.65 ^a^	555.76 ± 14.61 ^a^	567.24 ± 10.67 ^a^
**Total flavonoids**	172.42 ± 14.20 ^a^	99.52 ± 11.96 ^d^	119.04 ± 0.00 ^c^	125.40 ± 11.28 ^b,c^	123.29± 2.01 ^c^	142.02 ± 11.00 ^b^
**DPPH (IC_50_)**	8.21 ± 0.11 ^c^	12.56 ± 0.13 ^a^	9.38 ± 0.81 ^b^	7.17 ± 0.23 ^d^	5.54 ±0.03 ^e^	4.06 ± 0.16 ^f^
**ABTS (IC_50_)**	47.73 ± 0.36 ^c^	58.78 ± 0.46 ^a^	59.30 ± 0.84 ^a^	59.48 ± 0.55 ^a^	58.61 ±0.64 ^a^	56.21 ± 0.29 ^b^
**Betacyanin**	1970.00 ± 50.51 ^a^	ND	700.00 ± 10.76 ^d^	750.00 ± 0.00 ^d^	920.00 ± 30.82 ^c^	1090.00 ± 0.00 ^b^
**Betaxanthin**	1900.00 ± 50.93 ^a^	ND	500.00 ± 10.55 ^c^	440.00 ± 0.00 ^d^	480.00 ± 10.23 ^c^	590.00 ± 0.00 ^b^
**Betalain**	3860.00 ± 80.81 ^a^	ND	1200.00 ± 10.73 ^d^	1190.00 ± 0.00 ^d^	1410.00 ± 30.12 ^c^	1680.00 ± 80.11 ^b^

Data are presented as mean ± standard deviation (SD). Mean values in a row with different superscripts are significantly different at (*p <* 0.05). ND (not detected).

## Data Availability

The data presented in this study are available in this manuscript.
